# Supplementary material for: Integrated gene network analysis and experimental validation identify key hub genes in potato response to Potato Virus Y infection
Source: PLoS One. 2025 Aug 14;20(8):e0329747. doi: 10.1371/journal.pone.0329747 (PMC12352841; doi:10.1371/journal.pone.0329747)
Supplement: Attachment — Submitted filename: Response_to_reviewers_auresp_2.docx [file pone.0329747.s004.docx]

Reviewers' Comments to Author:

**Reviewer: 3**

1. Please do not start the Abstract or Introduction with The potato. Simply mention Potato

Response: Thank you for your comments. We removed “The”.

2. The references in text are somewhere names of authors and at some places numbered. Please make it uniform.

Response: We made all of them formatted with Plos One instructor.

3. Italicize cis in all cis-regulatory elements

Response: Done

4. Please mention the source(s) of miRNAs used in psRNATarget in lines 143-147

Response: We add a sentence to clear this section.

5. Line 310 enrichment analysis

Response: We corrected
